# Extracorporeal Hyperoxygenation Therapy (EHT) for Carbon Monoxide Poisoning: In-Vitro Proof of Principle

**DOI:** 10.3390/membranes12010056

**Published:** 2021-12-31

**Authors:** Niklas B. Steuer, Peter C. Schlanstein, Anke Hannig, Stephan Sibirtsev, Andreas Jupke, Thomas Schmitz-Rode, Rüdger Kopp, Ulrich Steinseifer, Georg Wagner, Jutta Arens

**Affiliations:** 1 Department of Cardiovascular Engineering, Institute of Applied Medical Engineering, Helmholtz Institute, Medical Faculty, RWTH Aachen University, Pauwelsstraße 20, 52074 Aachen, Germany; schlanstein@ame.rwth-aachen.de (P.C.S.); anke.hannig@rwth-aachen.de (A.H.); steinseifer@ame.rwth-aachen.de (U.S.); georg.wagner@rwth-aachen.de (G.W.); j.arens@utwente.nl (J.A.); 2 Fluid Process Engineering (AVT.FVT), RWTH Aachen University, Forckenbeckstraße 51, 52074 Aachen, Germany; stephan.sibirtsev@avt.rwth-aachen.de (S.S.); andreas.jupke@avt.rwth-aachen.de (A.J.); 3 Institute of Applied Medical Engineering, Helmholtz Institute, Medical Faculty, RWTH Aachen University, Pauwelsstraße 20, 52074 Aachen, Germany; smiro@ame.rwth-aachen.de; 4 Department of Intensive Care Medicine, Medical Faculty, RWTH Aachen University, Pauwelsstraße 30, 52074 Aachen, Germany; rkopp@ukaachen.de; 5 Department of Biomechanical Engineering, Faculty of Engineering Technology, University of Twente, De Horst 2, 7522LW Enschede, The Netherlands

**Keywords:** carbon monoxide, poisoning, extracorporeal therapy, hyperoxygenation, oxygenator, hollow fibre membrane oxygenator, bubble oxygenator

## Abstract

Carbon monoxide (CO) poisoning is the leading cause of poisoning-related deaths globally. The currently available therapy options are normobaric oxygen (NBO) and hyperbaric oxygen (HBO). While NBO lacks in efficacy, HBO is not available in all areas and countries. We present a novel method, extracorporeal hyperoxygenation therapy (EHT), for the treatment of CO poisoning that eliminates the CO by treating blood extracorporeally at elevated oxygen partial pressure. In this study, we proof the principle of the method in vitro using procine blood: Firstly, we investigated the difference in the CO elimination of a hollow fibre membrane oxygenator and a specifically designed batch oxygenator based on the bubble oxygenator principle at elevated pressures (1, 3 bar). Secondly, the batch oxygenator was redesigned and tested for a broader range of pressures (1, 3, 5, 7 bar) and temperatures (23, 30, 37 °C). So far, the shortest measured carboxyhemoglobin half-life in the blood was 21.32 min. In conclusion, EHT has the potential to provide an easily available and effective method for the treatment of CO poisoning.

## 1. Introduction

Carbon monoxide (CO) poisonings are responsible for estimated 50,000 emergency department visits and an estimated economic burden of $1.3 billion in the USA annually [1,2]. It is still the most lethal poisoning occurring in industrial nations nowadays, although the first extensive study on CO poisoning and its effects was published by Douglas et al. already in 1912 [3]. They ascertained that CO binds to hemoglobin (Hb), forms carboxyhemoglobin (CO-Hb), and thereby blocks the physiological oxygen (O_2_) pathway. Small amounts of CO were found to be sufficient to cause severe hypoxia, because the affinity of hemoglobin for CO is substantially higher than for O_2_ (230–270 times [4]). Subsequent studies discovered more disruptive effects of elevated CO concentrations: CO causes an increase in nitric oxide formation, leading to hypotension; CO has a negative impact on myocardial function by binding to myoglobin; CO binds to platelet hem protein and cytochrome c oxidase, causing direct cellular damage by interrupting cellular respiration. This leads to neuronal necrosis and apoptosis [5]. Furthermore, increased CO concentrations lead to the formation of reactive oxide species and inflammation [4,6,7]. The clinical symptoms range from headaches and nausea to visual disturbances and cardiac arrhythmia, and ultimately to coma, seizures, and breathing and circulatory failures [8]. Long-term effects are common, they occur in up to 40% of the patients. Most common are neurological sequelae but also dementia, psychosis, disturbance of memory and movement disorders have been reported [6,8].

**Figure 1:**
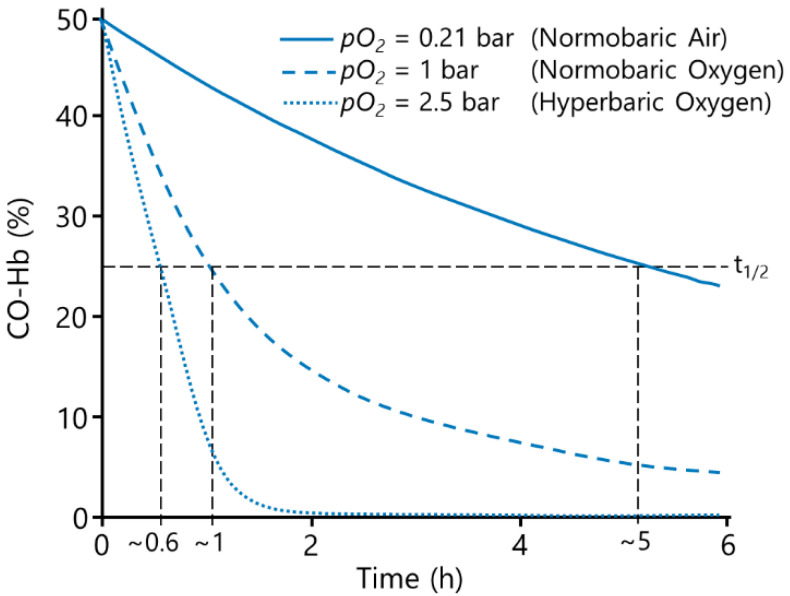
Influence of O_2_ partial pressure on CO-Hb-level decrease, determined from [5,9].

In room air, the half-life of carbon monoxide is approx. 320 min [5]. The current treatment of CO poisoning is normobaric oxygen (NBO) or hyperbaric oxygen (HBO), as the elimination of CO increases with increased O_2_ concentrations [4,9], see Figure 1. NBO is rapidly available and usually immediately initiated, decreasing the CO half-life to approx. 74 min [5]. HBO increases the physically solved O_2_ further, whereby the CO-Hb half-life is reduced drastically, resulting in a CO half-life of approx. 20 min [5]. The use of HBO compared to NBO was found to reduce the incidence of cognitive abnormalities from 33% to 18% [10]. However, while the rationale of the HBO treatment is compelling, up to date the use of HBO for the treatment of CO poisoning remains controversial, as there is no sufficient evidence of its benefits [9,11]. A recent review by Roderique et al. [7] even cautions for the use of HBO, as it further increases reactive oxygen species, potentially exacerbating the damage that is already occurring. Furthermore, in practice the limited number of hyperbaric chambers presents a major issue in therapy. A recent study by Chin et al. [12] concluded that only 11.9% of 361 surveyed hyperbaric centres in the USA were sufficiently equipped for dealing with emergency cases. Especially in remote areas the coverage is insufficient at best, resulting in transportation times of several hours to a hyperbaric chamber. Furthermore, the long preparation time of most available chambers further delays the rapidly needed therapy, even in metropolitan areas [5,9,13]. Additionally, a patient’s comorbidity such as, e.g., acute respiratory distress syndrome (ARDS) or chronic obstructive pulmonary disease (COPD) may mitigate the use of NBO and HBO, as the CO elimination takes place in the lungs [14,15]. Therefore, novel treatment options are needed [5].

Pharmacological treatment options that are currently being investigated include the use of a combination of hydroxocobalamin and ascorbic acid into a reduced form [16], a supramolecular complex (hemoCD) [17], and molecules based on bioengineered neuroglobin [18,19]. These drugs have shown a great affinity for CO and are used as scavenger molecules to eliminate carbon monoxide.

There are also non-pharmaceutical approaches, for example the ClearMate™ [20], which enhances the CO elimination by hyperventilation.

For many respiratory indications, such as COPD and ARDS, the use of extracorporeal membrane oxygenation (ECMO) has proven to be efficacious [21]. Within these systems, the native function of the lung, the uptake of oxygen and the removal of carbon dioxide, is taken over by an oxygenator [22]. As the elimination of CO takes place solely in the lungs, this approach could also be used for the cure of CO poisoning. However, ECMO is not designed for a rapid CO elimination. Therefore, the enhancement by phototherapy is investigated, whereby the dissociation of CO from hemoglobin is facilitated by light [23].

Our approach, the extracorporeal hyperoxygenation therapy (EHT) is based on applying the method of hyperbaric oxygenation to a gas exchanger, enhancing the elimination of CO by increasing the dissolved oxygen levels in the blood. As only the blood is affected, higher pressure levels can be reached than with HBO, because the patient is not exposed to the pressure. Additionally, an increase in reactive oxygen species would arise only there and not in the patient, as the half-life of most reactive oxygen species is in the range of micro or even nano seconds [24]. Moreover, the device could also be rather small and therefore portable. This would allow the start of the treatment directly at the site of the accident and also facilitate an area-wide, easily accessible coverage. Furthermore, patients suffering ARDS or COPD could be treated irrespective of the gas exchange performance of their lungs. Overall, we hypothesize that this approach provides a promising extension to the currently used treatment options.

To maximize the positive outcome of such a novel device, a high detoxification velocity is imperative. The CO elimination is presumably mainly depending on:

1. Velocity of chemical bonding processes;
2. Concentration of O_2_ and CO in plasma;
3. Shift of carbon monoxide-hemoglobin dissociation curve;
4. Diffusion of O_2_ to Hb and of CO away from Hb.

In order to examine the feasibility and limitations of the EHT regarding the detoxification velocity, we performed two series of experiments. Preliminary experiments comparing the performance of hollow fibre membrane oxygenators (HFMO) versus a specifically designed batch oxygenator (BO) based on the bubble oxygenator principle [25] were conducted. Subsequently, the more promising option was revised, and the performance regarding the detoxification velocity was tested for a broader range of operating points. Test variables were temperature and pressure within the device, which we assumed to be the primary influencing factors. As elevated temperatures and pressures in combination with blood-air contact can also be related to blood damage, the finally performed evaluation included the rate of hemolysis occurring during the detoxification.

## 2. Materials and Methods

### 2.1. Poisoning of Blood In Vitro

The poisoning of blood in vitro was carried out in a bench top circulation loop consisting of a cardiotomy reservoir, a roller pump (HL-20, Maquet, Rastatt, Germany), and a membrane oxygenator (hilite 7000, Xenios AG, Heilbronn, Germany). The circuit was filled with fully heparinized porcine blood from the slaughterhouse. Blood parameters were set to meet the requirements of ISO 7199 for blood-gas exchangers [26]. Blood flow was set to 3 L/min while sweep gas flow was 0.5 L/min containing 3% CO and 97% N_2_ (Linde AG, Pullach, Germany). When the CO-Hb-level reached the desired value, the poisoning was ended by stopping the blood flow and flooding the oxygenator fibres with pure nitrogen (N_2_). Afterwards, the CO-poisoned blood was filled into a blood bag and placed on a platform shaker to keep the blood homogenously mixed. The blood was always used at day of retrieval for all experiments.

### 2.2. Measured Parameters and Method of Analysis

The parameter of main interest was the fraction of CO-Hb in percent. It was measured using a blood gas analyser (ABL800 Flex, Radiometer Medical ApS, Brønshøj, Denmark), which was also used for measuring the pH-value.

Hemolysis was determined by photometric measurement of the plasma free hemoglobin (Ultrospec 2100 Pro, Biochrom, Berlin, Germany). The analysis of hemolysis was performed according to DIN 58,931 [27] by means of the cyanmethemoglobin method (Hemoglobin FS, DiaSys, Germany) according to manufacturers’ instructions. For this, the plasma of each blood sample was separated from the cells by double centrifugation at 1500× *g* for 15 min.

### 2.3. Preliminary Experiments

We hypothesized that using a specifically designed batch oxygenator (BO) based on the bubble oxygenator principle provides faster CO elimination than the hollow fibre membrane oxygenator (HFMO), due to the lack of mass transfer resistance associated with the membrane that inhibits a diffusion of CO out of the blood. Furthermore, the short residence time inside the HFMO is unfavourable because of the binding kinetics of CO to hemoglobin. Therefore, we performed preliminary experiments, comparing these two approaches for CO elimination: a previously described HFMO [28] was compared to the specifically designed BO.

#### 2.3.1. Test Set-Up Hollow Fibre Membrane Oxygenator

**Figure 2:**
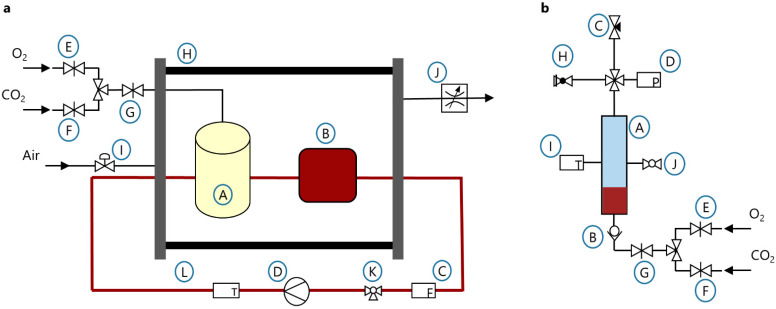
Experimental setups for the preliminary experiments (**a**) for the testing of a HFMO, (**b**) for the testing of a BO.

The experimental setup (Figure 2a) consisted of the HFMO (A), a blood bag (B), serving as a reservoir, a clamp-on flow sensor (C) (H9XL, Transonic Europe B.V., Elsloo, The Netherlands) connected to a flow meter (HT110, Transonic Europe B.V., Elsloo, The Netherlands), and a blood pump (D) (deltastream^®^ DP3, XENIOS AG, Heilbronn, Germany). The sweep gas of the oxygenator was controlled by mass controllers (E–G) (MASS-VIEW^®^ MV-304, Bronkhorst High-Tech B.V., AK Ruurlo, The Netherlands). For the experiments with excess pressure, the oxygenator and the blood bag were placed in a pressure resistant chamber (H), which was pressurized, using a pressure control valve (I) (VPPM-6L-L-1-G18-0L6H-V1P-S1C1, Festo GmbH and Co. KG, Esslingen, Germany). Due to the flexibility of the blood bag, the applied pressure was existent across the whole test loop. To avoid a pressure drop across the hollow fibre membrane, the sweep gas was pressurized to the identical pressure as the pressure resistant chamber, by the pressure control valve. A Series RM Rate-Master^®^ controller (J) (Dwyer Instruments Inc., Michigan City, IN, USA), connected to the chamber, was set to the same gas flow rate as the sweep gas, to prevent a pressure build-up inside the chamber. Samples were taken at the sampling port (K). The temperature was measured pre-oxygenator (L).

The test loop was primed with sterile 0.9% sodium chloride solution (B. Braun Melsungen AG, Melsungen, Germany) to wet the components. After draining the priming solution, the test loop was filled with 500 mL poisoned blood with a starting CO-Hb of ~30% (range: 29.5–32.7%) and 12 ± 1 g/dL of hemoglobin. The sweep gas was set to 5 L/min with a concentration of 5% carbon dioxode (CO_2_) and 95% O_2_. Thus, by maintaining a constant sweep gas flow throughout all experiments, the influence of the sweep gas flow on the CO elimination can be disregarded. The experiments were carried out at room temperature (Ø 24 ± 2 °C). Samples for the blood gas and hemolysis measurement were taken after 0 min, 5 min, 10 min, 15 min, and 30 min. The experimental matrix was set up by three different blood flow rates and two different pressure levels. The experiments were repeated two times. The test parameters are shown in Table 1.

#### 2.3.2. Test Set-Up Batch Oxygenator

The experimental setup can be seen in Figure 2b. A standard process engineering bubble column (A) (SCHOTT AG, Mitterteich, Germany) was used as a batch oxygenator. The blood inlet and a porosity 2 VitraPOR^®^ micro-immersion filter (ROBU^®^ Glasfilter-Geräte GmbH, Hattert, Germany) for gas dispersion and pressure build-up were located in the bottom of the column. The filter was connected via tubing and a check valve (B) to MASS-VIEW^®^ MV-304 controllers (E–G) (Bronkhorst High-Tech B.V., AK Ruurlo, The Netherlands), controlling the gas flow into the bubble column. A needle valve (C) to regulate the gas outflow was placed at the top of the column. Thus, by matching in- and outflow, a constant excess pressure could be maintained. The corresponding pressure sensor (D) (A-10, WIKA SE and Co. KG, Klingenberg, Germany) and a safety relief valve (H) (855811318001, ESSKA, Hamburg, Germany) were also connected to the top of the column. Furthermore, the top of the column was filled with filters from a cardiotomy reservoir (MVC 4030, XENIOS AG, Heilbronn, Germany) to prevent foam from rising. A temperature sensor (I) and a sampling port (J) were mounted in the middle of the column.

The BO was filled with 500 mL poisoned blood with a starting CO-Hb of ~30% (range: 29.7–33.5%) and 12 ± 1 g/dL of hemoglobin. For the pressure build-up, the gas flow was set to 0.1 L/min and the needle valve was closed until the operating pressure was reached (10 min). For experiments at ambient pressure, the same time was waited without gas flow. Then, the gas flow was increased to the respective test parameter. The needle valve was adjusted accordingly to maintain a constant operating pressure. Samples for the blood gas and hemolysis measurement were taken at the start of the CO elimination (0 min), at 15 min, and 30 min. The experimental matrix was set up by three different gas flow rates and two different pressure levels. The experiments were repeated three times. The test parameters are shown in Table 1.

### 2.4. Main Experimental Setup

**Figure 3:**
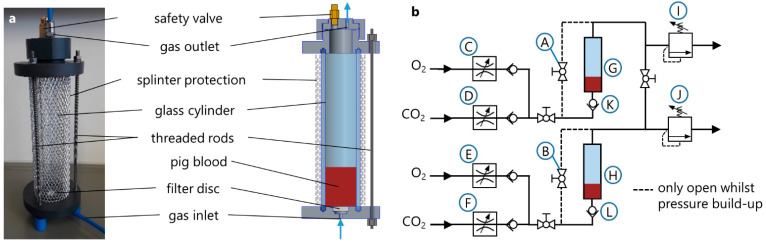
Structure of pressure-resistant BO (**a**) and main experimental setup for the operation of two BOs (**b**).

After the preliminary tests, the batch oxygenator was revised and scaled down to allow a temperature management and the operation at higher pressure levels, see Figure 3a. The main component of every BO was a 200 mm long cylinder of borosilicate glass with a diameter of 40 mm (Mennes, Selm, Germany). One glass cylinder was sealed between a bottom plate and a top plate serving as lids and was clamped with three threaded rods.

The bottom plate, made from PVC, served as inlet for the sweep gas and held a biplane filter disc, series 16, porosity 2, VitraPOR^®^ (ROBU^®^ Glasfilter-Geräte GmbH, Hattert, Germany) for gas distribution and bubble formation. The filter discs, which have pore sizes between 40 and 100 µm, were glued into the notches of the bottom plates with a solvent-free 2-component epoxy resin adhesive (UHU endfest 300, UHU GmbH and Co. KG, Bühl/Baden, Germany).

The top plate was also made from PVC and contained a connection panel, which held the gas outlet fitting and a safety valve. For safety reasons, an aluminium splinter protection was placed around the glass cylinder.

The pneumatic circuit for the operation of the BOs was realized by pneumatic fittings, tubing, and several ball valves (QH-QS-6, Festo GmbH and Co. KG, Esslingen, Germany) and check valves (H-QS-6, Festo GmbH and Co. KG, Esslingen, Germany). The circuit structure of two BOs, which were used in parallel for the testing of one operating point, is shown in Figure 3b. Two identical BOs (G, H) were used throughout the experiments. The gas flow was adjusted by four digital gas flow controllers (C–F) (ANALYT-MTC GmbH, Müllheim, Germany). In order to prevent any backwards flow of blood through the filter into the tubing system, check valves (K, L) (H-QS-6, Festo GmbH and Co. KG, Esslingen, Germany) were installed directly prior to the gas inlet of each BO. The two pressure regulators (I, J) (DVU01-100000, ESSKA, Hamburg, Germany) were used for controlling the pressures inside the BOs.

The temperature regulation of the blood inside the BOs was realized by a water bath, which was tempered by means of a temperature control unit E 100 (Lauda, Lauda-Königshofen, Germany). The two BOs were placed in the water bath during test runs.

### 2.5. Main Experimental Procedure

For preparation of the CO elimination, 50 mL of poisoned blood with an Hb-CO of ~42.5% (range: 41.3–44.8%) and 12 ± 1 g/dL of hemoglobin was drawn from the bag and filled in each BO. In order to prevent the formation of foam during the elimination, 50 µL of Antifoam 204 (Sigma Aldrich, St. Louis, MO, USA) were added to each BO by pipetting it directly onto the blood surface. After closing the BOs, both BOs were transferred into the already tempered water bath.

The pressure build-up in the BOs took place while the two ball valves (A, B) to the gas-outlet were open (Figure 3). This inhibited the gas passage through blood before the pressure had reached the target value. To keep the pH-value as constant as possible, a fixed amount of CO_2_ in the blood is desired. This was achieved by preconditioning the blood according to ISO 7199 and using a sweep gas with a defined CO_2_ concentration of 0.03 standard litre per minute.

The total gas volume flow (O_2_ plus CO_2_) inside the BOs was kept constant, regardless of the pressure level, and was set to 0.3 L/min per BO. Thereby, a constant influence of the gas flow rate on CO elimination and hemolysis was maintained.

The CO elimination was started when the pressure in the BOs had reached the target value. By closing the ball valves (A, B) to the gas-outlet, the gas was forced to pass through the check valves (K, L), the dispersers and into the blood in both BOs. Both BOs were set to identical pressure levels. Solely, the test duration was varied: for the first BO the elimination was stopped after 5 min, for the second BO after 15 min. Directly after turning off the gas supply of a BO, the pressure in the BO was released quickly. For the calculation of the CO-Hb half-life it was assumed that the measurements of both BOs are part of the same curve progression. By using two BOs with different termination times the blood was depressurized in the BOs and not the sample syringe, eliminating the problem of failed measurements due to bubbles in the samples.

Samples for the blood gas and hemolysis measurement were taken

- before CO elimination,
- shortly after filling of the BO with blood (0 min), and
- after CO elimination, directly after the pressure release at 5 or 15 min.

Three different examined temperatures of 23 °C, 30 °C and 37 °C and four pressure levels of 1 bar, 3 bar, 5 bar, and 7 bar absolute pressure set up the experimental matrix. This adds up to a total of 12 different test conditions carried out in this study. Every experiment was performed three times.

### 2.6. Calculations and Statistics

The CO-Hb level is measured using a blood gas analyser, which does not allow a continuous monitoring of CO-Hb, but only discrete measurement points. Therefore, it is difficult to poison the blood to exactly the same starting value CO-Hb_start_ for each series of tests. Hence, the absolute detoxification velocity (∆CO-Hb per minute) is not a suitable parameter for a comparison, since it is dependent on the starting value.

Pace et al. [29] described CO elimination to be following the simple exponential rate expression:

$$\text{CO-Hb}\left(t\right)={\text{CO-Hb}}_{\mathrm{start}}\times e^{-\mathrm{kt}}$$

CO-Hb(t) stands for the percentage of CO-Hb at time t. CO-Hb_start_ is the percentage before the CO elimination. The CO-Hb half-life is therefore a suitable parameter that describes the velocity of the CO elimination independent of the start value. The half-life was calculated using a nonlinear regression with the assumption that the plateau equals 0% CO-Hb. Subsequently, we performed a two-way ANOVA followed by Tukey’s multiple comparison test.

For each experiment, the plasma free hemoglobin (pfHb) was measured at the start and the end of the CO elimination. The values were subtracted to calculate the delta pfHb. Subsequently, we performed a two-way ANOVA followed by Tukey’s multiple comparison test.

To analyse the pH-value, an average value for each experiment was calculated, using the measurement at each time point. With these average values, we performed a two-way ANOVA followed by Tukey’s multiple comparison test. Zeitlicher verlauf.

All statistical analyses were performed with GraphPad Prism 9.

## 3. Results

### 3.1. Comparison of CO Elimination of HFMO and BO

**Figure 4:**
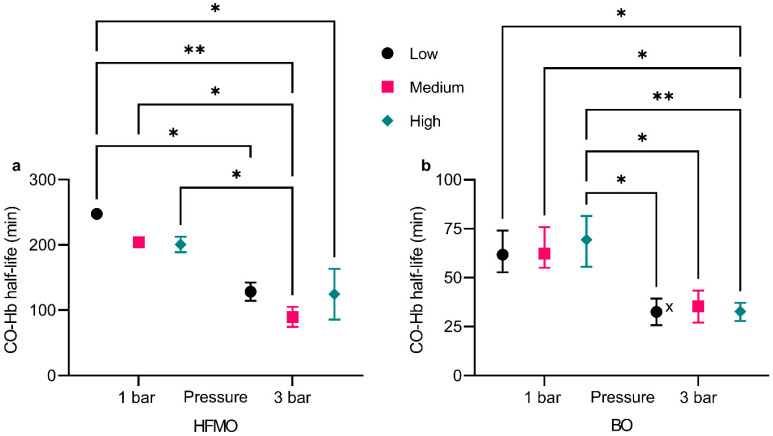
CO-Hb half-life with (**a**) HFMO and (**b**) BO dependent on pressure and flow rate. The whiskers represent the highest and lowest values of the corresponding experiments. Flow rate stands for blood flow rate for HFMO experiments and for gas flow rate for BO experiments. Note the different scale of the axes. (*) *p* ≤ 0.05, (**) *p* ≤ 0.01. (x) During one experiment, bubbles inside the samples prevented the analysis.

The CO-Hb half-life of the preliminary experiments are shown in Figure 4. The entire measurement data set is provided as supplementary information (Tables S1 and S2). Therein, failed measurements are identified. The characterization by blood gas analysis of some samples was impossible due to bubbles, which formed in the syringe during the release of excess pressure. Therefore, only intermediate samples were affected. The measurements of start and end samples were always successful.

In both the HFMO and the BO, a variation of the blood flow rate or the gas flow rate, respectively, does not affect the CO-Hb half-life significantly. An increase in pressure results in a faster CO elimination in both oxygenators, as expected. The overall effect is considered significant (*p* ≤ 0.0001). However, a superior performance of the specifically designed batch oxygenator, especially at an operating pressure of 3 bar, can be observed. The use of a BO leads to an increased performance by a factor of more than 3.5 compared to an HFMO, when comparing the averaged CO-Hb half-lives at a pressure of 3 bar. Furthermore, the increased pressure level entails a stronger effect on the half-life for the BO than for the HFMO. Based on these preliminary results, we decided to proceed using the BO, which we redesigned and evaluated in the main experiments.

### 3.2. Performance of Extracorporeal CO Elimination with a Revised Batch Oxygenator

**Figure 5:**
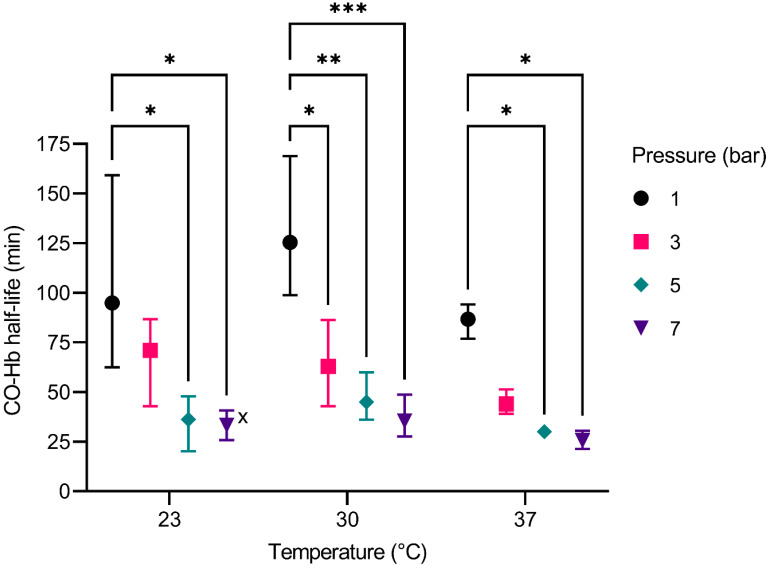
CO-Hb half-life of the revised BO dependent on temperature, and pressure. The whiskers represent the highest and lowest values of the corresponding experiments. (*) *p* ≤ 0.05, (**) *p* ≤ 0.01, (***) *p* ≤ 0.001. (x) One experiment was excluded due to extensive foam formation inside the BO.

Figure 5 shows the CO-Hb half-life in relation to different temperatures and varying pressure levels. The values were averaged from the results of three identical measurements. For the original data, see supplementary information (Table S3).

For the comparison of different pressures at constant temperatures it is notable that higher pressures result in a shorter half-life; the overall effect of the pressure is considered significant (*p* ≤ 0.0001). The increase from ambient pressure to 5 bar and 7 bar results in a significant decrease of the half-life for every temperature. The increase from ambient pressure to 3 bar is only significant at 30 °C. Additionally, the general decrease of the CO-Hb half-life seems to increase linearly with increasing pressures.

An increase in temperature, does not show a significant effect on the CO-Hb half-life. For an increase in temperature from 23 °C to 30 °C, for example, the half-life increases for a pressure of 1, 5, and 7 bar. Yet, for a pressure of 3 bar, a decrease of the half-life from 30 °C to 37 °C is visible. At the physiological temperature of 37 °C, the CO-Hb half-life reaches the lowest value at 37 °C and 7 bar. Finally, it is also notable that the variances of the measurements are distinctively lower at 37 °C.

### 3.3. Hemolysis

**Figure 6:**
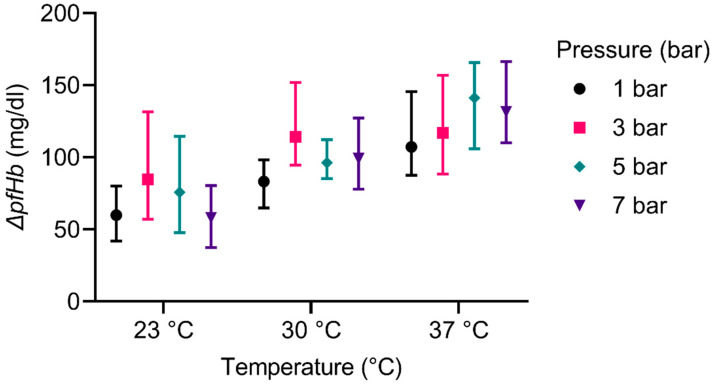
Plasma free hemoglobin dependent on temperature and pressure. The whiskers represent the highest and lowest values of the corresponding experiments.

The increase in plasma free hemoglobin during the experiments dependent on temperature and pressure is displayed in Figure 6. For the original data, see supplementary information (Table S4). The overall influence of the temperature is considered significant (*p* ≤ 0.001); experiments at higher temperatures generally result in higher concentrations of plasma free hemoglobin than experiments at lower temperatures. For experiments at 5 bar and 7 bar for example, the concentration of plasma free hemoglobin doubles with an increase in temperature from 23 °C to 37 °C. The overall effect of the pressure on the plasma free hemoglobin is considered not significant.

### 3.4. pH-Value

**Figure 7:**
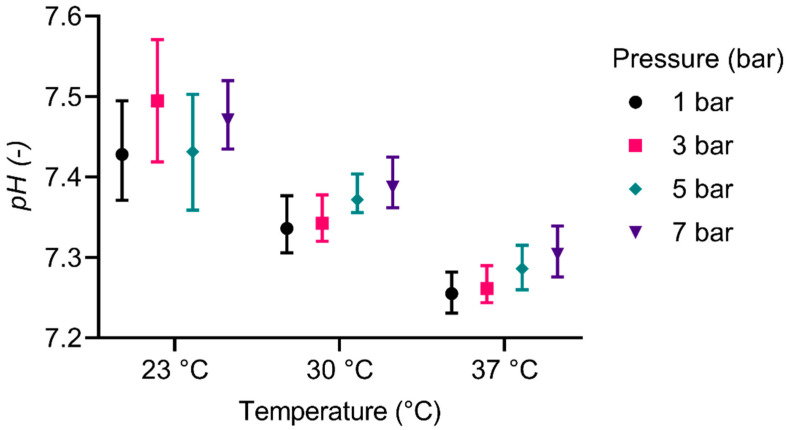
pH-value dependent on temperature, and pressure. The whiskers represent the highest and lowest values of the corresponding experiments.

Figure 7 shows the pH-values during the experiments dependent on temperature and pressure. For the original data, see Supplementary Information (Table S5). The results show high deviations, especially for lower temperatures, which decrease slightly at higher temperatures. For the experiments at 30 °C and 37 °C, increasing mean pH-values with increasing pressures can be observed. Overall, pH-values and temperature show a reciprocal relationship, which is considered significant (*p* ≤ 0.0001). The overall effect of the pressure is considered not significant.

## 4. Discussion

A novel treatment option for carbon monoxide poisonings is urgently needed, as demonstrated by the numerous research activities described in the introduction. However, the only approved therapy is the ClearMate, which has not gained clinical acceptance. The other approaches are still at the in-vivo stage, which means a failure rate of over 90% [30], in case of the pharmaceutical approaches. The light-enhanced ECMO is also a medical device with an extracorporeal approach. However, to achieve a pervasive illumination of the opaque blood, a vast surface area is required.

The overall aim of this study was to achieve a proof of principle of a novel therapeutical method that eliminates CO extracorporeally from blood. The underlying approach aims at enhancing the current therapies or at providing a novel therapy option where the current therapies are unavailable or of no avail. The novel method is realized by extracorporeal hyperoxygentation of the patient’s blood. The EHT allows for higher pressures than the HBO therapy, because the patient is not exposed to the pressure and therefore, no side effects such as oxygen toxicity etc. have to be considered. Furthermore, the resulting device could be small and portable, essentially bringing the HBO therapy to the patient.

In the first part of the study, the CO elimination performances of HFMO and BO were compared. The performance of a BO was increased by a factor of more than 3.5. Although the different flow rates, blood flow rate for the HFMO and gas flow rate for the BO lack in comparability, no influence on the performance is observable. Therefore, the comparison of the flow rates provides no bias towards the interpretation of the results.

We hypothesize that the mass transfer resistance due to membranes in the HFMO results in a slower CO elimination, compared to the BO. This is supported by the results of the preliminary experiments. Additionally important is the difference in residence time within HFMO and BO. On the one hand, the HFMO needs a continuous flow-through, to provide ample mixing of the blood. On the other hand, in the BO, the mixing is realized by the rising gas bubbles. It is therefore possible, to implement a batch process using a BO, thus providing higher residence times. This is especially important because of the binding kinetics: CO unbinds from hemoglobin at 1/2000 the speed of oxygen [31]. Therefore, a process with a higher residence time leads to a higher performance. Thus, the batch process of the utilized BO, which ensures a high residence time, might lead to the increased performance. The flow-through concept of the HFMO, while able to sufficiently oxygenate blood, does not provide enough residence time for an efficient elimination of CO. Hence, for the subsequent experiments, the concept of using a BO with a batch process was further investigated. Additionally, this supports our assumption of the velocity of the bonding processes as an influence on the CO elimination.

The overall effect of pressure on the elimination of CO has already been studied in vivo by numerous scientists working with HBO [5]. However, there has been no research concerning a practical approach with pressures higher than used in HBO therapy (2.5 bar–3 bar) and different temperatures, since such parameters cannot be safely investigated in vivo due to, e.g., oxygen toxicity. Nevertheless, our tests on the elimination of CO dependent on pressure and temperature provide evidence that a further increase in pressure and the alteration of the blood temperature might enhance the elimination of CO.

The decrease of the CO-Hb half-life due to increased pressure is shown by all experiments. This can be explained by Le Chatelier’s principle [32], by which the response of a stressed equilibrium system can be predicted. The basic concept of the elimination of CO from blood can be described as an equilibrium reaction, as first stated by Douglas et al. [3]:

$$O_{2}\text{-Hb}+\text{CO}⇌\text{CO-Hb}+O_{2}$$

here, the stress on the equilibrium system caused by the increased pressure manifests in two ways:

First, because the equilibrium of both sides of the reaction is dependent on the respective concentrations; an increase in O_2_ concentration in the plasma results in a shift of the equilibrium to the left side: more CO is unbound and dissolved in the plasma, which can then be eliminated. The increased O_2_ concentration is achieved by raising the overall pressure level and supplying high amounts of oxygen. This effect was seen in all our experiments, as an increase in pressure always led to an improved elimination of CO. The experiment at 23 °C and 5 bar, which showed a CO-Hb half-life of 7.72 min (see Supplementary Table S3), is regarded as an outlier. Overall, the effect is consistent with the experiences made with HBO [9].

Second, an increased pressure favours conditions that are less volumetric. In the literature, the activation volume of O_2_ binding to hemoglobin has been described as being positive, whereas the activation volume of CO binding to hemoglobin shows negative activation volumes. Therefore, as the pressure is increased, the equilibrium system experiences a stress that leads to the binding of more O_2_, as this state requires less volume [33,34,35]. This effect has a negative impact on the elimination of CO, yet it seems to be minor compared to the effect of the increased O_2_ concentration described before. An increase in pressure achieved with inert gases would probably make this effect more apparent but was not focus of our study.

Although the realistic operating temperature of our method would be 37 °C, we tested the CO elimination at a broader range of temperatures to determine the dependencies. In theory, varying the temperature during the CO elimination influences the elimination threefold:

First, with increasing temperature, the oxygen dissociation curve is shifted to the right, which decreases the affinity of hemoglobin for oxygen [36,37]. This effect is also true for CO, as the binding mechanism to the hemoglobin is similar [3,38]. The decreased affinity of hemoglobin for CO yields a faster CO elimination, whereas a decreased affinity for O_2_ results in a slower CO elimination. This is because of the lower affinity for O_2_, the chances of CO binding to hemoglobin are increased. However, unbound CO is eliminated from the plasma due to a concentration gradient towards the CO-free membranes or gas bubbles. O_2_ on the other hand is not eliminated, because the membranes are flushed with oxygen and the bubbles consist mainly of oxygen.

Second, a higher temperature facilitates diffusion, which becomes apparent in the Einstein-Smoluchowski relation [39,40]:

$$D=\frac{k_{B}\cdot T}{6\cdot \pi \cdot \eta \cdot R_{0}}$$

with k_B_ as Boltzmann constant, T as Temperature, η as viscosity, and R_0_ as particle radius. Thus, the mobility of CO and O_2_ in blood is increased, whereby the exit of CO out if the hem pocket and the subsequent diffusion towards the membranes and the gas bubbles is facilitated. For O_2_ on the other hand, this entails an easier diffusion into the hem pocket, once it is free from CO. Thereby, a rebinding of the CO is prohibited. Additionally, the reaction rate of O_2_ and hemoglobin is solely limited by the migration process of O_2_ through the Hb, as binding itself occurs so quickly [31,33]. The Einstein-Smoluchowski relation also implies that the diffusion is increased with lower viscosity of the liquid. In our case, the liquid is blood plasma, which shows a decrease in viscosity with higher temperatures, further enhancing the elimination of CO [41,42]. Furthermore, the rapid and uniform solution of O_2_ during the pressure build-up and thereby the increase in O_2_ concentration is also enhanced. This is favourable for the elimination of CO as well, because a high concentration (or partial pressure) of O_2_ yields the highest influence on the CO-Hb half-life (Figure 5).

Third and contrarily, higher temperatures lead to a lower solubility of gases in liquids. For blood, this was measured by Christoforides et al. [43], who ascertained that an increase of temperature from 23 °C to 37 °C entails a ca. 20% lower Bunsen solubility coefficient. This coefficient linearly correlates with the concentration of gas in the liquids. Therefore, the O_2_ concentration in the blood is reduced at high temperatures. This diminishes the effects of higher pressures on the CO-Hb half-life, compared to experiments at equal pressure levels but lower temperatures. The influence of the temperature on the Bunsen solubility coefficient is slightly regressive for increasing temperatures, which makes the reduction of the CO elimination less distinct.

In summary, the increase of the temperature yields opposing effects on the CO elimination. This may be the reason that the impact of the temperature shows no significant effect on the elimination of CO.

Bubble oxygenators, on which our revised design is based, are generally associated with high rates of hemolysis [44,45]. Pearson and McArdle [46] compared the hemocompatibility of several membrane and bubble oxygenators. The rise in plasma free hemoglobin (pfHb) caused by the cardiopulmonary bypass ranged from 13 mg/dl to 34 mg/dl for membrane oxygenators and from 9 mg/dl to 46 mg/dl for bubble oxygenators. The lowest measured values of pfHb in our study are ~60 mg/dl, the highest >140 mg/dl for a 15 min duration. Our presented results are also contradictory to the study of Bücherl [47], who reported that the hemolysis in a bubble oxygenator decreases with rising temperatures. These differences could be accounted to several factors: The design of our device was not focused on hemocompatibility, we even used non-hemocompatible materials such as borosilicate safety glass for the proof of principle. We also performed in-vitro experiments, whereas the cited articles studied the effects in vivo. Nakahara and Yoshida [48] studied the hemolysis in a bubble column in vitro and reported rates comparable to the results shown here. They also investigated the impact of antifoam agents on hemolysis and reported a dependency on the type and the concentration of antifoam. In our study, the increase of the temperature could entail an increased hemolytic activity and therefore be responsible for the deviation to Bücherl’s experiments. The pressure, contrarily, showed no distinct influence on the hemolysis, as seen in the results (Figure 6). This is in accordance with the literature [49,50,51,52,53]. Pohlmann et al. [54] even reported no impact of the air interface on the hemolysis at comparable ratios of blood volume and gas flow rates.

Even though it has been tried to maintain a constant CO_2_ partial pressure and therefore pH-value during all experiments by varying the CO_2_ fractions in the feed, the results indicate that this has not been successful (Figure 7). For an application in vivo, this is not tolerable and therefore further studies are needed. However, for the present study, the influence of the pH-value has to be analysed regarding the CO elimination. Most likely, the influence of temperature on the acid dissociation constant of the bicarbonate buffer system was not correctly considered. This could entail the decreasing pH-values with increasing temperatures. The pH-value is known to be of great importance for gas transport in blood. Lowering the pH-value leads to a right shift of the oxygen binding curve [36]. Since the binding of CO to Hb is similar to the one of O_2_ [3,38], a lower pH-value is therefore expected to accelerate the elimination of CO.

The in-vitro experiments were conducted two times for the HFMO and three times for each BO, as they were highly complex, especially for high pressure levels. Therefore, we present the entire data set in the results as well as in the supplementary information online. Nevertheless, the results proof the principle of extracorporeal elimination of CO at elevated pressure levels as aspired. For further development, the results can be interpreted as general tendencies, but more extensive studies still have to be conducted.

As discussed, the studied parameters influence the elimination of CO in many different, mostly opposing ways. From our experiments it is not possible to conclude the magnitude of the different influences. However, the focus of our study was to proof the principle of extracorporeal CO elimination using a novel concept. As of yet, no efforts have been made to optimize the CO elimination. For this subsequent optimization a more comprehensive study of the various influences is needed.

For a clinical applicability, not only the CO elimination has to be considered, but also the safety of the patient. With our concept, two main aspects result: First, the impact of the blood-air contact on the hemocompatibility. Bubble oxygenators have been associated with blood trauma [25] and are no longer clinically used. However, in our approach is has to be taken into account that, compared to approx. 6 h for bubble oxygenators, the blood is only treated for a very limited amount of time and then returned to the patient, where after a different volume of blood is drained and treated. The presented results regarding hemolysis seem promising considering that the device was not hemocompatibly designed. Nevertheless, other aspects of hemocompatibility, such as thrombogenicity and inflammatory reactions, have to be examined as well. Second, the device has to be designed in a way that the application of high pressures in the device does not endanger the patient or medical staff.

## 5. Conclusions

In conclusion, the presented experiments show that the extracorporeal elimination of CO from blood is possible. The shortest CO-Hb half-life at a physiological temperature of 37 °C was achieved at a pressure of 7 bar with a value of 20.4 min. This is in the range of the HBO therapy. However, because the filling and emptying, as well as the pressure build-up and release take time and as only a fraction of the whole blood can be treated at once, a further increase of the detoxification velocity is necessary. As mentioned, no effort has been made yet to optimize the device and therefore, the necessary improvement seems realistic. Following the redesign and optimization for detoxification velocity, and the validation of clinical safety, the presented concept has the potential to enhance the current therapy chain for CO poisoning by providing an exhaustive coverage more easily than HBO, and by offering a novel treatment option for paramedics in the field and physicians in the hospital.

## Figures and Tables

**Table 1:** Test parameters for the preliminary experiments.

HFMO					BO				
Blood Flow Rate (mL/min)			Pressure (bar)		Gas Flow Rate (L/min)			Pressure (bar)	
100 (low)	500 (medium)	1000 (high)	1	3	0.4 (low)	2.21 (medium)	4 (high)	1	3

## Data Availability

All data generated or analysed during this study are included in this published article (and its Supplementary Information files).

## Supplementary Materials

The following supporting information can be downloaded at: https://www.mdpi.com/article/10.3390/membranes12010056/s1, Table S1: Entire data set for the CO elimination with the HFMO; Table S2: Entire data set for the CO elimination with the BO; Table S3: Entire data set for the CO elimination with the revised BO; Table S4: Entire data set for the hemolysis of the experiments with the revised BO; Table S5: Entire data set for the pH-value of the experiments with the revised BO.
