# Supplementary material for: Extracorporeal Hyperoxygenation Therapy (EHT) for Carbon Monoxide Poisoning: In-Vitro Proof of Principle
Source: Membranes (Basel). 2021 Dec 31;12(1):56. doi: 10.3390/membranes12010056 (PMC8779470; doi:10.3390/membranes12010056)
Supplement: Supplementary file 1 [file membranes-12-00056-s001.zip › membranes-1496122-supplementary.pdf]

Supplementary Materials

Extracorporeal Hyperoxygenation Therapy (EHT) for Carbon Monoxide Poisoning: In-Vitro Proof of Principle

Niklas B. Steuer [1,*], Peter C. Schlanstein [1], Anke Hannig [1], Stephan Sibirtsev [2], Andreas Jupke [2], Thomas Schmitz-Rode [3], Rüdger Kopp [4], Ulrich Steinseifer [1], Georg Wagner [1] and Jutta Arens [1,5]

[1] Department of Cardiovascular Engineering, Institute of Applied Medical Engineering, Helmholtz Institute, Medical Faculty, RWTH Aachen University, Pauwelsstraße 20, 52074 Aachen, Germany; schlanstein@ame.rwth-aachen.de (P.C.S.); anke.hannig@rwth-aachen.de (A.H.);
steinseifer@ame.rwth-aachen.de (U.S.); georg.wagner@rwth-aachen.de (G.W.); j.arens@utwente.nl (J.A.)

[2] Fluid Process Engineering (AVT.FVT), RWTH Aachen University, Forckenbeckstraße 51, 52074 Aachen, Germany; stephan.sibirtsev@avt.rwth-aachen.de (S.S.); andreas.jupke@avt.rwth-aachen.de (A.J.)

[3] Institute of Applied Medical Engineering, Helmholtz Institute, Medical Faculty, RWTH Aachen University, Pauwelsstraße 20, 52074 Aachen, Germany; smiro@ame.rwth-aachen.de (T.S.-R.)

[4] Department of Intensive Care Medicine, Medical Faculty, RWTH Aachen University, Pauwelsstraße 30, 52074 Aachen, Germany; rkopp@ukaachen.de

[5] Department of Biomechanical Engineering, Faculty of Engineering Technology, University of Twente, De Horst 2, 7522LW Enschede, The Netherlands

* Correspondence: steuer@ame.rwth-aachen.de; Tel.:+49-241-80-88764

Table S1: Entire data set for the CO elimination with the HFMO

Sample			CO-Hb (%)		CO-Hb half life (min)	
p (bar)	Flow rate	t (min)	n = 1	n = 2	n = 1	n = 2
		0	30.1	31.2		
		5	28.5	30.9		
		10	28.9	30.3		
		15	28.2	29.8		
1	low	30	27.3	28.8	243.3	252.1
		0	29.9	31.4		
		5	28.8	30.5		
		10	28.3	30		
		15	27.8	29.7		
1	medium	30	26.8	28.3	199.1	209.5
		0	30	31.1		
		5	28.7	30.2		
		10	28.3	29.8		
		15	27.7	29.3		
1	high	30	26.7	28.1	188.9	212.7
		0	31.6	31.5		
		5	30.1	30.6		
		10	28.9	28.7		
		15	28.8	28.5		
3	low	30	27.1	26.3	142.3	114.4
		0	30.1	31.4		
		5	29.5	28.8		
		10	26.7	28		
		15	25.8	27.9		
3	medium	30	23.1	25.3	74.28	105.2
		0	32.1	31.3		
		5	29.9	30.3		
		10	28.7	29.4		
		15	28.6	28.9		
3	high	30	24.8	27.5	85.61	163.4

Table S2: Entire data set for the CO elimination with the BO

Sample			CO-Hb (%)			CO-Hb half-life (min)		
p (bar)	Flow rate	t (min)	n = 1	n = 2	n = 3	n = 1	n = 2	n = 3
		0	32.4	32.8	31			
		15	27.4	25.2	24.6			
1	low	30	24.6	23.5	21.1	74.02	58.52	52.68
		0	32	32.8	31.1			
		15	26.9	26.1	25			
1	medium	30	24.5	22.9	21.5	75.82	56.04	55.08
		0	32.6	33.3	31			
		15	26.7	N/A	24.5			
1	high	30	24.6	25.8	21.6	71.04	81.49	55.51
		0	29.4	30.1	N/A			
		15	24.1	N/A	N/A			
3	low	30	16.8	13.4	N/A	39.25	25.7	N/A
		0	29.6	25.7	26.8			
		15	26.1	N/A	N/A			
3	medium	30	15	11.9	16.6	35.43	27.01	43.41
		0	29.2	30.9	27.4			
		15	20.9	21.5	N/A			
3	high	30	13.5	18.3	14.6	27.9	37.16	33.03

Table S3: Entire data set for the CO elimination with the revised BO

Sample			CO-Hb (%)			CO-Hb half-life (min)		
p (bar)	T (°C)	t (min)	n=1	n=2	n=3	n=1	n=2	n=3
		0	43.1	41.5	44.8			
		5	41.7	40.4	43			
1	23	15	40.3	35.3	38	159.2	62.99	62.53
		0	43	41.5	44.4			
		5	41.8	40.4	43.2			
1	30	15	39.1	37.4	41.7	108.70	98.80	168.90
		0	43	41.3	44.5			
		5	41.6	39.7	43.3			
1	37	15	38.3	36.1	39.9	89.2	76.85	94.11
		0	43.1	41.5	44.5			
		5	40.9	39.6	42.2			
3	23	15	33.9	36.6	39.4	42.95	83.22	86.70
		0	43.1	41.3	44.4			
		5	41.2	37.9	42.2			
3	30	15	33.9	34.6	39.3	42.86	59.75	86.25
		0	42.9	41.3	44.5			
		5	39.9	38.4	42.2			
3	37	15	32.9	32.2	36.4	39.05	41.66	51.41
		0	43.1	41.4	44.5			
		5	39	36.8	41.6			
5	23	15	25.5	33.2	34.5	20.18	47.91	40.68
		0	43.1	41.3	44.4			
		5	39.7	38.6	41.6			
5	30	15	33	31	37.3	38.89	36.10	59.94
		0	43	41.4	44.3			
		5	39.3	37.5	41			
5	37	15	30.8	28.6	31.7	31.14	28.14	31.04
		0	43.1	41.4	44.5			
		5	38.7	36.6	40.9			
7	23	15	12	32	29.7	(9,94)	40.73	25.84
		0	42.9	41.3	44.3			
		5	39.3	36.7	41.4			
7	30	15	29.4	29.1	35.8	27.58	29.7	48.74
		0	43	41.3	44.3			
		5	39.2	35.1	39.6			
7	37	15	26.2	27.1	31.5	21.32	24.61	30.49

Table S4: Entire data set for the hemolysis of the experiments with the revised BO

Sample			pfHb (mg/dl)		
p (bar)	T (°C)	t (min)	n = 1	n = 2	n = 3
		0	20.7	27.49	25.505
		5	52.07	64.51	66.5
1	23	15	62.575	107.65	83.265
		0	24.455	30.915	26.15
		5	59.55	72.365	70.825
1	30	15	111.05	95.905	124.475
		0	30.56	31.475	25.875
		5	74.765	82.595	76.67
1	37	15	118.9	177.033	113.5
		0	23.445	28.595	24.405
		5	45.605	88.745	75.85
3	23	15	80.45	160.1	89.62
		0	23.81	30.005	27.2
		5	65.505	81.77	76.53
3	30	15	175.65	126.15	121.7
		0	27.255	33.515	27.715
		5	79.13	107.9	77.64
3	37	15	184.1	139.45	116.05
		0	24.565	29.43	26.025
		5	52.72	71.48	69.8
5	23	15	72.295	144	91.155
		0	27.865	31.12	27.51
		5	78.32	85.93	78.83
5	30	15	118.95	116.3	139.75
		0	27.77	36.575	30.06
		5	86	86.61	78.915
5	37	15	133.65	188.75	195.75
		0	24.165	33.46	26.91
		5	44.075	73.82	70.65
7	23	15	61.5	113.85	83.625
		0	25.525	31.32	25.355
		5	68.97	73.47	77.8
7	30	15	152.7	123.45	103.3
		0	30.045	34.935	28.175
		5	74.99	96.695	91.05
7	37	15	196.45	145	146.25

Table S5: Entire data set for the pH-value of the experiments with the revised BO

Sample			pH (-)		
p (bar)	T (°C)	t (min)	n = 1	n = 2	n = 3
		0	7.844	8.042	7.841
		5	7.256	7.285	7.250
1	23	15	7.013	7.159	7.166
		0	7.635	7.801	7.650
		5	7.146	7.191	7.176
1	30	15	7.138	7.139	7.152
		0	7.468	7.602	7.496
		5	7.104	7.113	7.128
1	37	15	7.120	7.132	7.136
		0	7.818	8.010	7.824
		5	7.721	7.271	7.240
3	23	15	7.175	7.204	7.193
		0	7.619	7.784	7.638
		5	7.194	7.184	7.191
3	30	15	7.146	7.166	7.162
		0	7.467	7.587	7.487
		5	7.139	7.136	7.113
3	37	15	7.125	7.146	7.154
		0	7.766	7.986	7.806
		5	7.256	7.261	7.256
5	23	15	7.053	7.261	7.233
		0	7.608	7.765	7.626
		5	7.270	7.239	7.193
5	30	15	7.190	7.208	7.250
		0	7.446	7.576	7.477
		5	7.172	7.175	7.154
5	37	15	7.161	7.194	7.218
		0	7.756	7.971	7.787
		5	7.269	7.299	7.299
7	23	15	7.280	7.289	7.288
		0	7.587	7.749	7.614
		5	7.309	7.241	7.214
7	30	15	7.191	7.285	7.294
		0	7.449	7.564	7.474
		5	7.204	7.218	7.132
7	37	15	7.174	7.235	7.287
